# Immune snapshots along the inflammation-to-cancer road in bladder urothelium

**DOI:** 10.3389/fimmu.2025.1685237

**Published:** 2025-11-03

**Authors:** Lingxiang Lu, Fei Wang, Zhenfan Wang, Shuai Guo, Minjun Jiang, Zheng Ma

**Affiliations:** ^1^ Department of Urology, Suzhou Ninth People’s Hospital, Soochow University, Suzhou, Jiangsu, China; ^2^ Department of Urology, The Affiliated Suzhou Hospital of Nanjing Medical University, Suzhou Municipal Hospital, Gusu School, Nanjing Medical University, Suzhou, Jiangsu, China

**Keywords:** bladder cancer, inflammation-to-cancer transition, immune microenvironment, immune checkpoint, IFN-γ signature, tumor immunology, spatial transcriptomics, liquid biopsy

## Abstract

The inflammatory microenvironment formed by chronic inflammation is not only a major risk factor for cancer but also a well-recognized precursor to bladder cancer. However, the immunological transitions that occur along the inflammation-to-cancer continuum remain incompletely understood. This mini-review synthesizes recent advances in understanding how the immune microenvironment evolves from an inflamed yet non-malignant urothelium to invasive carcinoma. First, we discuss how persistent stimuli—such as chronic infection or exposure to carcinogens—disrupt immune homeostasis, leading to sustained interferon signaling, cytokine secretion, and immune cell infiltration. Second, during preneoplastic and dysplastic stages, the immune landscape gradually shifts toward an environment enriched in regulatory T cells and characterized by dysfunctional cytotoxic T cells. Furthermore, in established tumors, immune evasion is primarily driven by T cell exhaustion, myeloid cell–mediated immunosuppression, and fibroblast-associated immune exclusion. Finally, advances in spatial transcriptomics, single-cell technologies, and urinary exosomal profiling have enabled precise “immune snapshots” of these transitions, providing new avenues for biomarker development and therapeutic strategy selection. Mapping these dynamic immune states holds great promise for improving risk stratification, facilitating early detection, and enabling personalized immunotherapy, ultimately translating immune snapshots into actionable strategies for bladder cancer prevention and treatment.

## 1 Introduction

Bladder cancer remains one of the most prevalent and recurrent malignancies worldwide, with urothelial carcinoma accounting for over 90% of cases (1). Its development is frequently preceded by chronic inflammation, which may arise from recurrent urinary tract infections, exposure to carcinogens such as tobacco smoke and aromatic amines, or intravesical instillation therapies like Bacillus Calmette-Guérin (BCG) (2). Bladder cancer (BC) is a highly immunogenic tumor, and immunotherapy plays a central role in its management, particularly in non–muscle-invasive disease (NMIBC) (3). Among these approaches, Bacillus Calmette–Guérin (BCG)—a live attenuated Mycobacterium bovis strain introduced in 1976—remains a cornerstone treatment (4). Delivered intravesically, BCG activates both innate and adaptive immune responses: it recruits macrophages, dendritic cells, and T lymphocytes to the bladder mucosa and induces the release of pro-inflammatory cytokines such as IFN-γ, TNF-α, and interleukins, which together enhance cytotoxic T cell activation (5). BCG may also upregulate immune checkpoints, boosting immune recognition of tumor cells. These coordinated responses enable effective elimination of primary tumors and potential metastases, underscoring BCG’s pivotal role in NMIBC therapy (6).

Mounting evidence supports the paradigm that inflammation not only accompanies tumorigenesis but can serve as a driver of malignant transformation by reshaping the tissue microenvironment and inducing genetic instability (7). In the bladder urothelium, persistent inflammation triggers a cascade of immunological events—recruitment of innate immune cells, cytokine secretion, epithelial stress responses—that collectively disturb homeostasis and promote oncogenic reprogramming (8). These events form the early stages of a continuum known as the “inflammation-to-cancer transition,” wherein immunological cues evolve in parallel with histopathological changes from normal epithelium to dysplasia, carcinoma *in situ* (CIS), and invasive cancer (9). However, while histological staging is well defined, the immunological landscape across this progression remains incompletely mapped (10).

Understanding the immune contexture at discrete phases of bladder tumorigenesis is essential for both early detection and rational therapy design (11). Immune cell composition, cytokine profiles, and checkpoint molecule expression can differ vastly between inflamed but non-malignant urothelium and established tumors (12). Capturing these differences—through what we term “immune snapshots”—can provide insights into the immunological tipping points that govern the transition from defense to tolerance, and ultimately, to escape (13). Emerging technologies such as spatial transcriptomics, single-cell RNA sequencing, and exosomal profiling have enabled unprecedented resolution in delineating these immune states (14). Gene signatures reflective of interferon signaling, myeloid skewing, or T cell dysfunction have already demonstrated prognostic and predictive value in bladder cancer cohorts (15). Likewise, animal models of chemically induced cystitis or urothelial carcinoma offer valuable platforms for temporal tracking of immune evolution (16).

In this mini-review, we synthesize current knowledge on the immune microenvironment along the inflammation-to-cancer spectrum in the bladder. We highlight key immune shifts, introduce the concept of phase-specific immune signatures, and discuss how such snapshots may inform biomarker development, risk stratification, and immunotherapy strategies.

## 2 Chronic inflammation and the urothelial immune landscape

**Figure 1:**
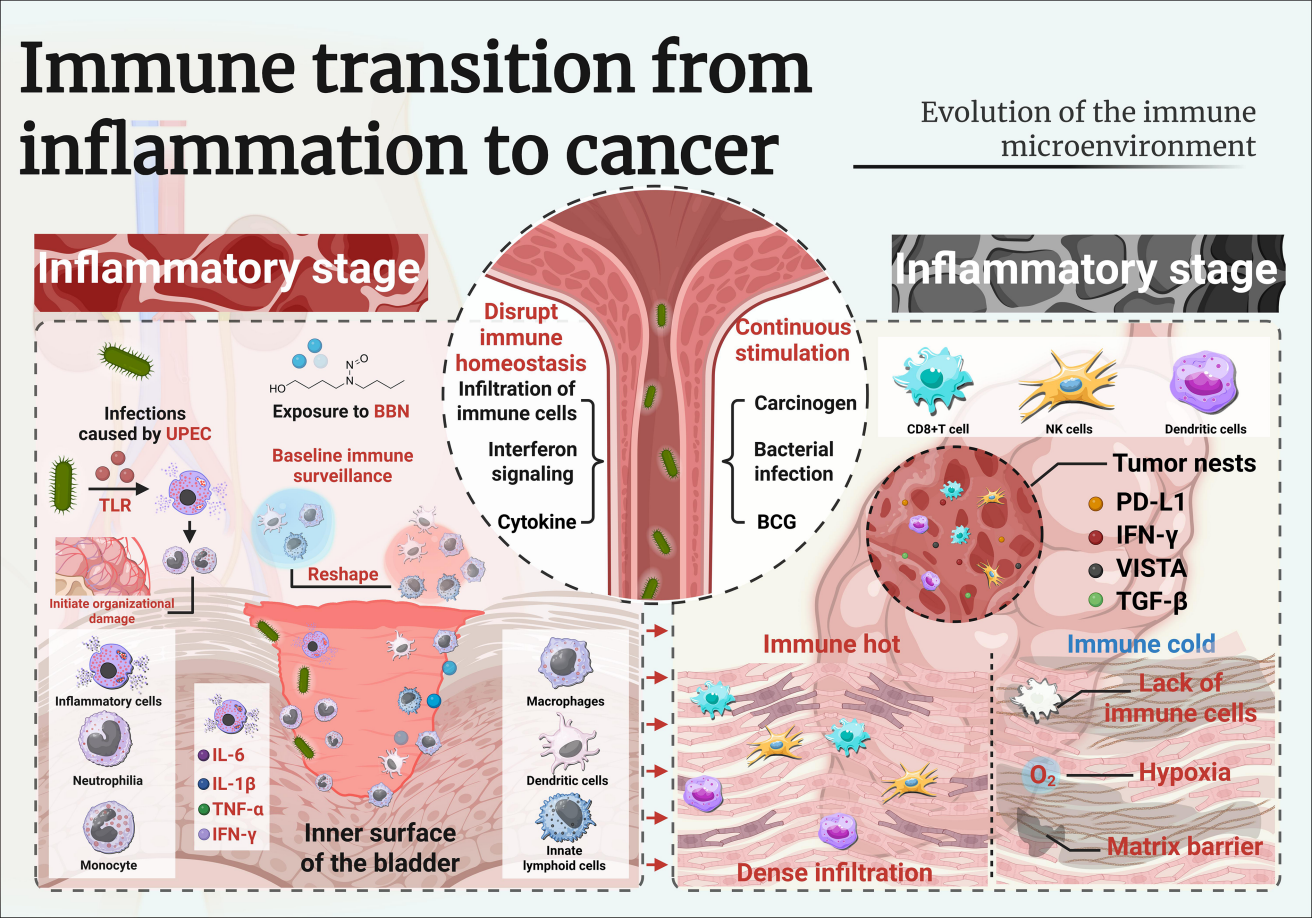
Imunune transition from inflammation to cancer.

The urothelium, a highly specialized transitional epithelium lining the bladder, is normally characterized by a quiescent immune environment (15). Baseline immune surveillance is maintained by tissue-resident macrophages, dendritic cells, and innate lymphoid cells, while adaptive immune activity remains limited under homeostatic conditions (16). However, upon exposure to chronic inflammatory stimuli—such as bacterial infection, chemical carcinogens (e.g., N-butyl-N-(4-hydroxybutyl)nitrosamine [BBN]), or repeated trauma—this balance is disrupted, leading to a profound remodeling of the local immune milieu (17) (**Figure 1**).

### 2.1 Triggers of chronic inflammation in the bladder

Recurrent urinary tract infections (UTIs), especially those caused by uropathogenic Escherichia coli (UPEC), are among the most common triggers of chronic bladder inflammation (18). Upon infection, bacterial components are recognized by Toll-like receptors (TLRs) on urothelial cells, activating downstream signaling cascades that stimulate the release of proinflammatory cytokines such as IL-6, IL-1β, TNF-α, and IFN-γ (19, 20). These cytokines, in turn, recruit neutrophils and monocytes to the bladder wall, initiating cycles of tissue injury and repair which, if sustained, can become maladaptive and promote pathological changes.

Experimental evidence from rodent models further supports this progression. Chronic exposure to N-butyl-N-(4-hydroxybutyl)nitrosamine (BBN) in drinking water recapitulates the stepwise transition from persistent inflammation to malignancy observed in human bladder carcinogenesis (21). Early disease stages are characterized by the infiltration of myeloid cells—particularly Ly6C^+^ monocytes and F4/80^+^ macrophages—accompanied by fibroblast activation and increased production of reactive oxygen species (ROS) (22). Together, these changes establish a “pre-tumor niche” that fosters genomic instability, epithelial hyperplasia, and ultimately, neoplastic transformation (23).

### 2.2 Immune cell dynamics in chronically inflamed urothelium

Chronically inflamed bladder mucosa demonstrates enhanced infiltration of both innate and adaptive immune cells (24). Neutrophils dominate the early response, releasing elastase and neutrophil extracellular traps (NETs), which can cause DNA damage in epithelial cells (25).

Over time, monocyte-derived macrophages and dendritic cells accumulate, shaping antigen presentation and polarization of T helper responses (26). Several studies have documented increased CD4^+^ T cell and regulatory T cell (Treg) populations in inflamed urothelium, suggesting an attempt to regulate excessive inflammation (27). As inflammation persists, monocyte-derived macrophages and dendritic cells accumulate and shape antigen presentation, leading to increased numbers of CD4^+^ T cells and regulatory T cells, while sustained interferon signaling upregulates antigen processing machinery and immune checkpoint molecules (28).

### 2.3 Cytokine and chemokine signatures

Transcriptomic analyses of chronically inflamed bladder tissue identify a conserved cytokine milieu characterized by IL-6, CXCL1, CXCL8, and IFN-γ (25). These mediators not only amplify immune cell recruitment but also modulate epithelial plasticity and promote epithelial–mesenchymal transition (EMT), a key step toward dysplasia and invasion (26). Chemokines such as CXCL9/10/11, typically associated with T cell attraction, are paradoxically expressed in regions where cytotoxic T cells are scarce, indicating the presence of immune exclusion mechanisms (27).

The role of IL-6/STAT3 signaling is particularly well documented in both human samples and BBN-treated mice (28). STAT3 activation in urothelial cells promotes proliferation and survival, while also skewing immune responses toward an immunosuppressive myeloid phenotype. This dual effect reinforces the chronic inflammatory loop and sets the stage for immune escape (23). The mechanism of chronic inflammation mediated by immune cells and their secreted cytokines is detailed in **Table 1**.

**Table 1:** Immune components and cytokines in chronically inflamed bladder urothelium.

Immune component	Function in chronic inflammation	Key molecules
Neutrophils	Early recruitment; ROS and NETs production	MPO, ELANE, CXCL1
Monocytes/Macrophages	Antigen presentation, cytokine secretion, M2-like immunosuppression	IL-10, TNF-α, CD163, ARG1
Dendritic Cells	Maturation impaired under chronic inflammation	CD11c, CD86, CCR7
CD4^+^ T Cells	Promote inflammation or regulatory feedback	IFN-γ, IL-17, FOXP3 (Tregs)
Urothelial cells	Cytokine release, checkpoint induction	IL-6, CXCL8, PD-L1, VISTA, MHC-I/II

### 2.4 Lessons from BCG and cystitis models

Controlled inflammatory responses induced by Bacillus Calmette-Guérin (BCG) therapy present a contrasting model (21). BCG-induced cystitis triggers robust IFN-γ-mediated responses and recruits effector T cells, generating an immunogenic environment that differs from the tolerogenic, carcinogen-induced setting (29, 30). Recurrent UTIs and chemical exposures like BBN initiate chronic bladder inflammation by activating innate immune pathways and shaping a complex cytokine milieu. This inflammatory environment influences immune cell dynamics and ultimately determines the balance between tumor surveillance and immune escape.

## 3 Immune rewiring in preneoplastic and dysplastic states

Immune Rewiring in Preneoplastic and Dysplastic States.

As inflammation persists, the bladder urothelium undergoes progressive architectural and molecular changes that precede overt malignancy (31). These intermediate stages—ranging from reactive atypia and hyperplasia to dysplasia and carcinoma *in situ* (CIS)—are not merely passive histological transitions but are accompanied by active immune rewiring (32). The immune microenvironment in these phases is distinct from both homeostatic and tumor states, marked by paradoxical features of activation and suppression (33).

### 3.1 IFN-γ–driven epithelial responses and checkpoint induction

Sustained IFN-γ signaling in preneoplastic urothelium, triggered by activated T cells or BCG‐induced inflammation, leads to a robust transcriptional upregulation of antigen presentation machinery, including MHC class I and II molecules (e.g., HLA-A, HLA-DRB) and proteasomal components like TAP1 and PSMB9 (34–36). *In vitro* exposure of normal urothelial cells to IFN-γ similarly induces key immune checkpoint molecules, notably PD-L1 and VISTA, suggesting that immune editing is initiated well before overt neoplastic transformation and may function to limit tissue damage, but under chronic antigenic stimulation, this process can promote a tolerogenic, tumor-permissive microenvironment (28, 37–39).

### 3.2 Spatial and single-cell insights: heterogeneity emerges early

Recent advances in spatial transcriptomics and single-cell RNA sequencing have revealed striking heterogeneity within dysplastic urothelium (40). A subset of epithelial cells acquire proinflammatory transcriptional programs (e.g., IRF1, STAT1), while others upregulate immune evasive signatures (e.g., TGF-β, CTLA-4 ligands) (41). This co-existence suggests an early divergence in epithelial-immune cross-talk, possibly influenced by local cytokine gradients or stromal interactions (42).

Immune cell populations in dysplastic lesions also become more diverse. While CD4^+^ T cells and macrophages persist, an influx of regulatory T cells, exhausted CD8^+^ T cells (expressing PD-1, LAG-3), and immature dendritic cells has been observed in both human CIS biopsies and BBN-induced lesions in mice (36). These cells exhibit low cytotoxic activity but high expression of immunoregulatory genes, suggesting a shift toward immune tolerance (38).

### 3.3 IFN-γ gene signature and early prognostic value

A 33-gene IFN-γ response signature derived from *in vitro* stimulated urothelial cells has been shown to stratify patient outcomes in non-muscle invasive bladder cancer (NMIBC) (39). High expression of this signature is associated with longer recurrence-free survival, suggesting that robust early immune activation may predict better immunosurveillance (40).

However, this benefit appears context-dependent. In muscle-invasive bladder cancer (MIBC), high IFN-γ signatures are paradoxically linked to immune checkpoint upregulation and therapeutic resistance (41). Thus, the timing, duration, and localization of interferon signaling must be interpreted within the evolving immune landscape (42).

### 3.4 Chemokine-checkpoint paradox and immune exclusion

Another hallmark of dysplastic immune rewiring is the “chemokine-checkpoint paradox”: high levels of T cell–attracting chemokines (CXCL9, CXCL10, CXCL11) are present, but effective cytotoxic T cell infiltration is limited (31). This may result from stromal or epithelial expression of checkpoint molecules (PD-L1, TIM-3 ligands), aberrant vasculature, or fibroblast-mediated immune exclusion (32).

In BBN models, lesions exhibiting strong CXCL9 expression paradoxically display sparse CD8^+^ T cell infiltration, despite the chemotactic gradient (33). These findings imply that chemokine production alone is insufficient for immune recruitment unless checkpoint-mediated barriers are removed (34). IFN-γ signaling exerts a dual role in early immunosurveillance and later immune evasion, underscoring the need for therapeutic strategies that overcome checkpoint barriers while harnessing early inflammatory signals. Addressing this balance is crucial for improving outcomes in bladder cancer.

## 4 Immune microenvironment in established bladder cancer

As urothelial dysplasia progresses to carcinoma *in situ* and ultimately invasive bladder cancer, the immune landscape undergoes profound transformation (**Figure 1**) (43). The shift from a chronically inflamed but immunologically active microenvironment to one characterized by dysfunction and tolerance is a hallmark of tumor immune escape (44). In established bladder tumors, the immune contexture reflects a dynamic balance between residual anti-tumor immunity and dominant immunosuppressive mechanisms that enable tumor growth and therapeutic resistance (44).

**Figure 1**. Immune rewiring in preneoplastic and dysplastic states of bladder urothelium.

### 4.1 Spatial distribution and composition of tumor-infiltrating immune cells

Recent investigations underscore that the immune microenvironment in established bladder cancer is highly heterogeneous, with spatial transcriptomics revealing distinct niches. Tumor-infiltrating lymphocytes (TILs) are frequently present in bladder tumors, but their density, composition, and spatial arrangement vary significantly across patients and tumor regions (45). “Immune hot” tumors exhibit dense infiltration of CD8^+^ T cells, natural killer (NK) cells, and antigen-presenting dendritic cells (DCs), often localized at the invasive margin or within tumor nests (46). In contrast, “immune cold” tumors lack significant immune cell presence and may be associated with stromal barriers, hypoxia, or poor antigenicity (47). Spatial transcriptomic studies have revealed that even within the same tumor, immune cells may segregate into peritumoral, stromal, or intratumoral niches, each governed by distinct cytokine and chemokine networks (48). For instance, intratumoral regions may express high levels of IFN-γ–responsive genes and checkpoint molecules (e.g., PD-L1, VISTA), while stromal zones are enriched with suppressive myeloid populations and fibroblast-derived TGF-β (49).

### 4.2 T cell dysfunction and checkpoint expression

Although CD8^+^ cytotoxic T cells are present in many bladder tumors, their functional capacity is often impaired (50). These cells exhibit features of exhaustion, characterized by sustained expression of PD-1, LAG-3, and TIM-3, diminished production of granzyme B and IFN-γ, and altered metabolic profiles (e.g., mitochondrial dysfunction, lipid accumulation) (51), which together result in diminished effector functions and cytotoxic capacity. The co-expression of multiple immune checkpoints, including PD-L1, VISTA, and TIGIT, suggests a highly regulated suppressive environment (52).

Based on the quantity and activity of T cells, further classification can be carried out. Molecular profiling has revealed at least two major immune-related subtypes of bladder cancer: 1) T-cell inflamed subtype: Enriched with IFN-γ signature, high TIL density, increased expression of PD-L1 and other checkpoints; typically more responsive to immune checkpoint blockade (ICB). 2) Immune desert or myeloid-dominant subtype: Characterized by poor T cell infiltration, high MDSC/TAM burden, and dominant TGF-β/IL-10 signaling; often resistant to ICB. These immune phenotypes correlate with molecular subtypes of bladder cancer (e.g., luminal, basal, neuroendocrine) and have implications for therapy selection (47, 53, 54).

### 4.3 Immunosuppressive myeloid cells and fibroblasts

Tumor-associated macrophages (TAMs), particularly the M2-like subtype (CD163^+^, ARG1^+^), dominate the myeloid compartment in advanced bladder cancer (49). These cells produce IL-10, TGF-β, and prostaglandin E2 (PGE2), suppressing T cell activation and promoting tumor cell proliferation (50). Similarly, myeloid-derived suppressor cells (MDSCs) inhibit both innate and adaptive immunity through arginase activity, reactive oxygen species (ROS), and nitric oxide production (51).

Cancer-associated fibroblasts (CAFs) contribute to immune exclusion by remodeling the extracellular matrix and secreting CXCL12, which forms a physical and chemokine-mediated barrier to T cell infiltration (51). In some bladder tumors, CAF-rich regions are virtually devoid of effector T cells, despite high chemokine expression, a phenomenon also observed in pancreatic and prostate cancers (48).

### 4.4 Predictors of response to immunotherapy

Checkpoint inhibitors targeting PD-1 or PD-L1 (e.g., atezolizumab, nivolumab) have demonstrated clinical benefit in a subset of bladder cancer patients, particularly those with high tumor mutational burden (TMB), pre-existing TILs, and elevated IFN-γ signatures (46). The IMvigor210 trial stratified patients by immune phenotype and found that responders tended to have T cell–inflamed tumors with high expression of PD-L1 on immune cells (IC2/3) (49). Conversely, non-responders often exhibited high myeloid signatures and TGF-β–driven exclusion patterns (50). Efforts to improve response rates now focus on rational combinations: ICB plus chemotherapy, anti-TGF-β agents, CSF1R inhibitors (targeting TAMs), or intravesical agents that modulate the tumor immune microenvironment (51).

Checkpoint inhibitors targeting PD-1 or PD-L1, such as atezolizumab and nivolumab, have shown significant clinical efficacy in a subset of bladder cancer patients, particularly those characterized by a high tumor mutational burden (TMB), pre-existing tumor-infiltrating lymphocytes (TILs), and elevated IFN-γ–associated gene signatures (46). Evidence from the IMvigor210 clinical trial further supports this observation: patients who responded to therapy typically exhibited T cell–inflamed tumor phenotypes, marked by high PD-L1 expression on immune cells (IC2/3) (49). In contrast, non-responders frequently displayed immunosuppressive microenvironments, characterized by dominant myeloid gene signatures and TGF-β–mediated immune exclusion, which hinder effective T cell infiltration and limit therapeutic efficacy (50). To overcome these resistance mechanisms and improve response rates, current strategies focus on rational combination therapies. These include combining immune checkpoint blockade (ICB) with chemotherapy, anti–TGF-β agents, CSF1R inhibitors that target tumor-associated macrophages (TAMs), or intravesical immunomodulatory agents designed to remodel the tumor immune microenvironment and enhance antitumor immunity (51).

## 5 Clinical and translational outlook: snapshots as biomarkers

**Figure 2:**
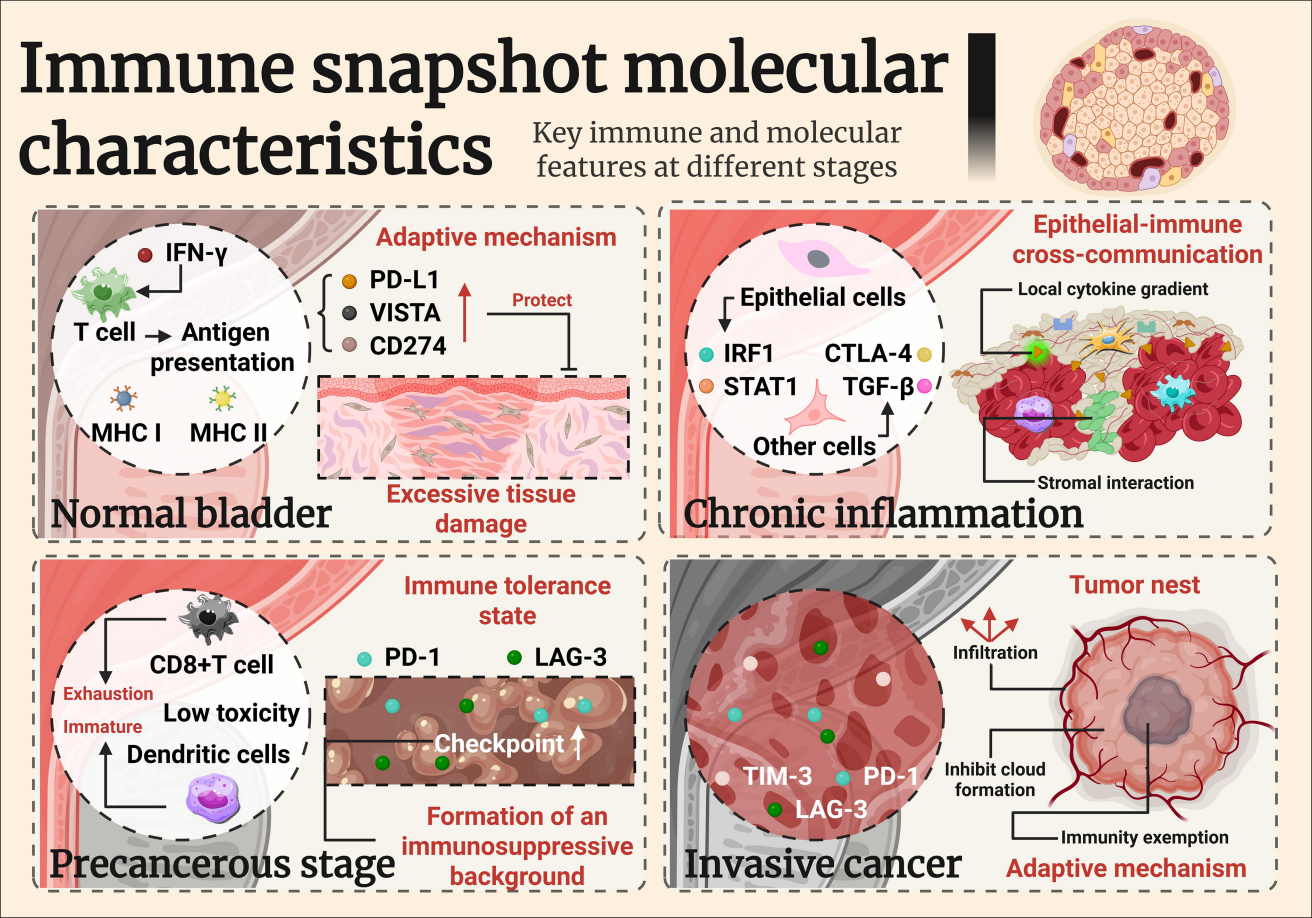
Imumune snapshot molecular characteristics.

The concept of immune “snapshots” along the inflammation-to-cancer continuum offers a framework not only for understanding tumorigenesis, but also for identifying actionable biomarkers, therapeutic windows, and strategies for patient stratification. As technologies for immune profiling evolve, there is growing potential to incorporate temporal and spatial immune signatures into clinical decision-making (55)(**Figure 2**).

### 5.1 Early detection and risk stratification

Phase-specific immune signatures—such as IFN-γ–responsive gene panels, checkpoint expression patterns, or T cell infiltration profiles—may serve as early indicators of neoplastic transformation (56). For instance, a high IFN-γ signature in non-malignant but inflamed urothelium could predict effective immune surveillance and a lower risk of progression (57) in **Table 2**. Conversely, the emergence of suppressive myeloid markers or checkpoint co-expression may signal immune escape and imminent tumorigenesis (58).

**Table 2:** Immune snapshots as predictive biomarkers in bladder cancer.

Immune snapshot	Predictive implication	Example applications
IFN-γ signature high	Better prognosis in NMIBC; possible ICB responsiveness	33-gene IFN-γ score, IMvigor210 responder subset
PD-L1/VISTA co-expression	Immune suppression; candidate for dual checkpoint blockade	IHC or RNAseq-based stratification
TGF-β–rich stroma	Immune exclusion; resistance to ICB	Use of anti-TGF-β in combination trials
CXCL9–11 high, T cell exclusion	Chemokine-checkpoint paradox; need for ECM targeting	Predicts failure of monotherapy ICB
Urinary exosomal PD-L1/IFNG	Liquid biopsy biomarker for immune activation	Potential surveillance marker post-BCG

These insights open the door for longitudinal immune monitoring in patients with chronic bladder inflammation, recurrent cystitis, or prior cancer history (59). In the future, patients could be risk-stratified not only by histopathology but by dynamic immunological states, enabling more personalized surveillance intervals or early interventions (60).

### 5.2 Liquid biopsy and non-invasive immune profiling

Urine-based assays represent an attractive and non-invasive approach to capturing immune snapshots in bladder cancer (60). Urinary exosomes, shed by urothelial cells and infiltrating immune cells, contain mRNA, miRNA, and protein cargo reflective of the tumor microenvironment (61). Several studies have demonstrated that urinary exosomal PD-L1, IFN-γ–inducible genes, or TCR/BCR repertoire fragments can mirror intratumoral immune activity (62).

Beyond exosomes, circulating immune cells, cytokines, and tumor-derived DNA (ctDNA) in plasma may also reflect bladder tumor immunity, especially in advanced disease (59). High-throughput immune repertoire sequencing, combined with AI-assisted pattern recognition, is being explored to identify immune “fingerprints” predictive of recurrence or therapeutic response (63).

### 5.3 Predicting and enhancing response to immunotherapy

As checkpoint inhibitors become standard in both non-muscle invasive and advanced bladder cancer, the need for robust predictive biomarkers becomes critical (61). Immune snapshots offer a more nuanced alternative to single-parameter markers such as PD-L1 immunohistochemistry or TMB (62). For example: 1) T cell–inflamed snapshots (high CXCL9–11, granzyme B, IFNG, CD8A) are predictive of response to PD-1/PD-L1 inhibitors (64). 2) Myeloid-dominant or TGF-β–rich snapshots are associated with resistance and may benefit from ICB plus stromal-targeting therapies (62). 3) Mixed phenotypes may require combination approaches involving chemotherapy, BCG, or targeted agents. Importantly, the spatial context matters: immune cells excluded from the tumor core but present in the periphery (“immune-excluded” phenotype) may require normalization of vasculature, ECM, or fibroblast networks before immunotherapy becomes effective (63).

### 5.4 From bench to bedside: clinical implementation challenges

The implementation of immune snapshot–based biomarkers is hindered by a lack of uniformity in immune cell phenotyping, spatial analysis, and gene expression quantification protocols, which vary significantly across platforms and studies (65). Current spatial transcriptomics (ST) technologies remain at a relatively early stage of development and are evolving rapidly, yet they still require trade-offs between spatial resolution, transcriptome coverage, and detection sensitivity. Spatial proteomics currently provides coverage far below that of the full proteome, although new approaches may eventually enable direct protein sequencing within tissues (66). To obtain a complete molecular landscape, additional modalities are required. Spatial genomics (67), epigenomics (68), and metabolomics (69) methodologies are under active development, but their integration with ST or proteomic data remains a major challenge. For example, spatial genomics may offer valuable insights into the role of somatic mutations in aging and age-associated immune senescence. Notably, the recently developed Slide-tags technique enables *in situ* labeling of individual cells with 10 μm spatial barcodes, followed by nuclei isolation for single-nucleus RNA sequencing (70). This innovation allows the direct transfer of single-cell sequencing design principles to spatially resolved multimodal data acquisition, thereby achieving true single-cell resolution. These inconsistencies contribute to challenges in data comparability and reproducibility, underscoring the need for harmonized protocols and centralized standard operating procedures. Moreover, sampling bias driven by tumor heterogeneity can underrepresent key immune populations in small or spatially restricted biopsies, limiting the precision of snapshot-based diagnostics (71, 72). In terms of clinical translation, current immune landscapes and molecular phenotypes can be integrated into prospective clinical trials and risk models by applying advanced methodologies such as multiplex imaging, single-cell sequencing, and automated data analysis platforms, which enable the construction of detailed immunological profiles that correlate with clinical outcomes (73, 74). These approaches can inform patient stratification and therapeutic decision-making by capturing dynamic shifts in the immune microenvironment, particularly when coupled with longitudinal sampling strategies. However, limitations such as variability in urine biomarker detection and spatial sampling constraints must be systematically addressed through multicenter collaborative efforts that standardize preanalytical and analytic processes (75). Immune snapshot–based biomarkers hold promise for personalized treatment strategies. Their successful clinical integration depends on rigorous harmonization and the development of robust methodologies to overcome sampling bias and inherent tumor heterogeneity.

### 5.5 Future directions

Cancer-associated fibroblasts (CAFs) are central mediators of tumor progression and immune evasion, acting through secretion of cytokines (e.g., IL-6) and modulation of the extracellular matrix to impair immune cell infiltration (76, 77). Emerging methodologies to overcome CAF-mediated immune exclusion include targeted immunotherapies such as FAP-specific adoptive T cell treatments and CAR T cell strategies, as well as vaccine approaches aimed at enhancing T-cell responses and reducing immune tolerance. Advanced techniques, including single-cell transcriptomics and spatial transcriptomics, are being deployed to resolve CAF heterogeneity and identify precise molecular markers (78). Additional strategies focus on reprogramming CAFs toward a quiescent phenotype using inhibitors like those targeting NADPH oxidase-4 or TGF-β, which in turn can enhance the efficacy of immune checkpoint inhibitors (79, 80). Imaging modalities like [68Ga]Ga-FAPI-46 PET/CT also contribute to assessing desmoplasia and predicting metastatic risk (81). Further research must define specific CAF subpopulations and their molecular signatures to enable precise targeting while minimizing systemic toxicity (82). Establishing standardized detection methods that preserve *in vivo* CAF phenotypes and integrating multi-omic analyses will be critical. Additionally, combinatorial treatment approaches that concurrently modulate CAF functionality and augment antitumor immunity are imperative for advancing cancer therapy (77). Targeted immunotherapies and advanced molecular profiling are essential next steps in overcoming CAF-mediated immune exclusion. Optimizing combinational approaches will be key to enhancing overall treatment efficacy.

## 6 Conclusion

The transition from inflammation to cancer in the bladder urothelium is a dynamic, immune-driven continuum in which evolving immune signals mirror histological and molecular changes. Each stage—from innate immune cell infiltration during chronic inflammation to the formation of immunosuppressive niches in invasive cancer—offers a distinct “immune snapshot” that reveals mechanisms of immune control, adaptation, and eventual tumor promotion. Advances in spatial transcriptomics, single-cell sequencing, and liquid biopsy now allow these states to be profiled with high resolution, guiding early biomarker discovery, risk stratification, and personalized immunotherapy. Integrating multi-omics data with AI and conducting longitudinal studies will further refine immune classification systems, enabling tailored interventions and improved outcomes.
